# Clearance of extracellular human amyloid-β aggregates in *C. elegans* by nutraceutical and pharmaceutical interventions

**DOI:** 10.17912/micropub.biology.000907

**Published:** 2024-01-08

**Authors:** Arastu Sharma, Collin Y Ewald

**Affiliations:** 1 Eidgenössische Technische Hochschule Zürich, Department of Health Sciences and Technology, Institute of Translational Medicine, 8603 Schwerzenbach-Zürich, Switzerland; 2 Johns Hopkins University, Baltimore, Maryland, United States

## Abstract

Numerous anti-amyloid therapies have seen recent clinical development and approval, such as the monoclonal antibodies aducanumab and lecanemab. However, in Alzheimer’s disease patients, amyloid-β (Aβ) plaques are found embedded in the extracellular matrix and surrounded by collagens, which might hinder these antibodies from targeting the plaques. We reasoned that various different nutraceutical and pharmaceutical agents might induce collagen and extracellular matrix turnover and removal of these collagen-embedded amyloid-β (Aβ) plaques. To address this idea, here, we used a transgenic *C. elegans* strain, LSD2104 , expressing fluorescent human Aβ _1-42_ as an *in-vivo* model for secreted amyloid aggregation in the extracellular matrix. We performed a screen of various nutraceuticals and pharmaceuticals along with different combinations, and we found that quercetin 350 µM and rifampicin 75 µM successfully cleared the extracellular amyloid plaque burden compared to the 0.2% DMSO control group, with a combination of the two agents producing the maximum effect compared to either drug alone. These results may implicate the exploration of combination therapeutics of nutraceuticals and pharmaceuticals in the clearance of amyloid-β (Aβ) plaques in Alzheimer’s disease.

**Figure 1. Clearance of Extracellular Human Amyloid-Beta (Aβ) Aggregates in C. elegans by Nutraceutical and Pharmaceutical Interventions:**
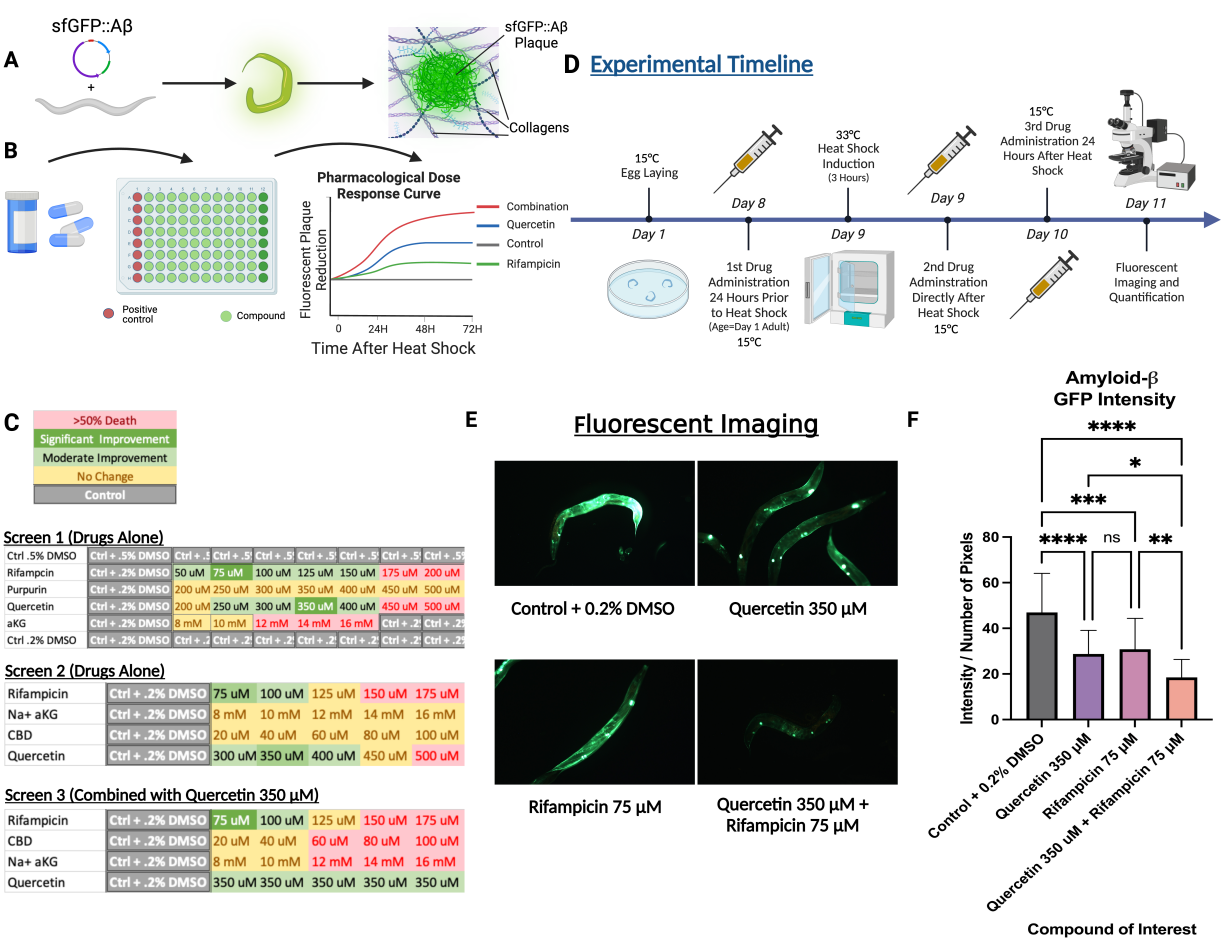
A) Depiction of the transgenic strain LSD2104 with the transgene sfGFP::Aβ _1-42_ under control of heat shock protein *hsp-16.2* promoter. Upon 3 hours of 33°C heat shock, amyloid-beta (Aβ) induction occurred, forming aggregates in the extracellular space over 24 hours. For details, see Materials and Methods. B) Depiction of screening for drug effects on amyloid aggregation, as measured through fluorescence intensity. Results informed various dosages for the efficacy of aggregate removal. C) Results of drug screens on amyloid aggregate removal, with color-coded legend depicting the efficacy of a given intervention. D) Depiction of experimental timeline for egg laying, amyloid induction, drug administration, and fluorescent imaging. LSD2104 *C. elegans* were maintained at 15°C, and heat shock was performed at 33°C for 3 hours. E) Fluorescent images of successful drug candidates with strong aggregation evident in the cuticle ECM of the transgenic LSD2104 *C. elegans* and coelomocytes. F) Intensity over the number of pixels (I/N) after quantification of each drug group against control, indicating the amount of amyloid burden. Statistical analysis between the geometric mean of groups was performed using one-way ANOVA, with bars representing geometric SEM and a significance threshold of (P<0.05). The combination of quercetin 350 µM and rifampicin 75 µM showed superiority in reducing aggregate burden compared to either drug alone, and both drugs administered separately reduced the amyloid burden compared to the control. Additional trials and data are found in Extended Data Figures 1-2, and Source Data File 1.

## Description

Aggregation of amyloid-β (Aβ) is considered one of the main hallmarks of Alzheimer’s disease (Perneczky et al., 2023) . However, despite amyloid aggregation being the widely accepted theory of disease pathology, many anti-amyloid therapies have failed to survive the obstacle of clinical trial validation, with lack of efficacy or toxicity being the main culprits of trial failure (Dai et al., 2022; Karran & De Strooper, 2022; Xie et al., 2022) . Currently approved drugs include the human immunoglobulin G1 antibody aducanemab (Grover & Jain, 2022) , and lecanemab is the most recent to receive FDA approval for the treatment of Alzheimer’s disease (Levin et al., 2022; Shi et al., 2022; van Dyck et al., 2023) . However, Aβ aggregates in the extracellular space may be blocked by various ECM components, such as heparan sulfate proteoglycans (HSPGs) (Gupta-Bansal et al., 1995; van Horssen et al., 2003) . These ECM components may potentially hamper the access to and breakdown of the Aβ plaques by antibody therapies, complicating their therapeutic outcomes (Alavi Naini & Soussi-Yanicostas, 2018) , and the disruption of proteoglycan and Aβ interaction has been shown to prevent amyloid-related pathology (Horssen et al., 2003). Here, we explore other therapeutic options to promote the clearance of these Aβ plaques embedded within the ECM.

Various pharmaceuticals and nutraceuticals have been shown to extend lifespan, such as metformin or quercetin, but their roles in age-related diseases are being explored further (Chen et al., 2017.; Liao et al., 2022; Saul et al., 2008; Zu et al., 2021) . Nutraceuticals have been tested in *C. elegans* models of Alzheimer’s disease to ameliorate disease pathology (McCarty, 2006) . Plant extracts, such as *Withania somnifera* and *Centella asiatica,* have been shown *in vitro* to prevent amyloid fibrillation and resulting plaque formation (Witter et al., 2018) , and ursolic acid has been shown to upregulate the proteasome in *C.* *elegans* to enhance the endogenous reduction of Aβ levels *in vivo* (Rigacci & Stefani, 2015; Wang et al., 2022) . Quercetin exhibits marked anti-inflammatory, antioxidant, and anti-amyloid, and acetylcholinesterase inhibitory characteristics in numerous *in-vivo* and *in-vitro* studies (Khan et al., 2019) . Furthermore, drug synergies may modulate or compound beneficial molecular pathways, resulting in enhanced therapeutic outcomes, as demonstrated previously in *C. elegans* (Admasu et al., 2018) . The conserved pathways between humans and *C. elegans* involving amyloid dynamics require further elucidation, particularly when translating anti-amyloid therapeutics to the clinical sphere (Ewald and Li, 2010; Apostolakou et al., 2021; Park et al., 2023) .

Previously, certain ECM proteins were shown to alter the level of extracellular amyloid aggregate burden, directly implicating the role of the ECM in the maintenance and removal of Aβ, particularly with the knockdown of *dyp-3* and *col-8* collagens (Jongsma et al., 2023) . Metalloproteinase A Disintegrin and Metalloproteinase 2 ( ADM-2 ) overexpression alone was also shown to be effective in clearing the Aβ burden embedded extracellularly in the LSD2104 strain of *C. elegans* (Jongsma et al., 2023) . In accordance with the previously performed induction of GFP-tagged human Aβ in the LSD2104 strain (Jongsma et al., 2023) , we induced sfGFP::Aβ _1-42_ expression and secretion with 3 hours of heat shock ( **Figure 1A** ). We established a screening protocol of drugs that was performed using 24-well and 96-well plates with varying concentrations of compounds, the number of doses of compounds, and experimental timelines (24H vs. 48H after heat shock) to determine the optimal dosage schedule. We performed a literature search identifying 39 compounds meeting our inclusion criteria ( **Extended Data Table 2** ). The inclusion criteria were: 1. The compound had been shown to increase the lifespan of an organism, 2. The compound had any implications for alleviating Aβ burden in experimental models, and 3. The compound has been linked to reducing neuroinflammation and improved cognition in experimental models of Alzheimer’s disease. Out of the 39 compounds, we decided to screen 15 compounds based on their relative novelty (underexplored) and potential feasibility for application in *C. elegans* ( **Extended Data Table 2** ). Out of the 15 compounds, 5 compounds affected the amyloid load in the *C. elegans* ECM ( **Figure 1B** ).

Screening results of various drugs indicated optimal dosages and combinations, with quercetin 350 µM and rifampicin 75 µM producing the largest visible reduction in amyloid burden. Higher dosages of drugs resulted in extensive death of *C. elegans* with rifampicin 175-200 µM, quercetin 450-500 µM, and alpha-ketoglutarate (aKG) 12-16 mM ( **Figure 1C, Screen 1** ). No difference was observed between DMSO 0.2% and DMSO 0.5%. The screen was repeated using Na ^+^ aKG instead of Ca ^2+^ aKG due to the excess deaths that occurred at higher dosages, likely due to the acidity of Ca ^2+^ aKG. Na ^+^ aKG and Cannabidiol (CBD) failed to produce improvement, but rifampicin and quercetin exhibited a reduction in visible Aβ fluorescent intensity ( **Figure 1C, Screen 2** ). A combination screen was performed, and the strongest visible reduction in Aβ was with the two optimal doses of rifampicin 75 µM and quercetin 350 µM. Excessive death was observed in higher concentrations in the combination screen, likely due to the increased toxicity of simultaneous drug administration ( **Figure 1C, Screen 3** ).

We wanted to confirm whether the action of the drug was affecting the induction, formation, or clearance of Aβ aggregates. Induction of the sfGFP::Aβ _1-42_ was evident at 24 hours; however, there was no statistically significant reduction of fluorescence in experimental groups with drug administration 24 hours before and directly after heat shock ( **Extended Data Figure 1A** ) or a single drug administration directly after heat shock ( **Extended Data Figure 1B** ). Fluorescence was significantly reduced between 24H and 48H after heat shock of the quercetin group (P<0.0001), implying that drug administration did not affect the induction level nor aggregation of sfGFP::Aβ _1-42_ , but rather affected clearance of the secreted sfGFP::Aβ _1-42_ ( **Extended Data Figure 1C** ). Taken together, the experimental timeline of imaging 48H-post heat shock produces the most vibrant fluorescent imaging and optimal clearance of the amyloid aggregates, with drug administration 24H before, directly after, and 24H after heat shock ( **Figure 1D** ).

Next, to validate the screening results, we assessed sfGFP::Aβ _1-42_ aggregation levels after 48 hours post-induction. Fluorescent images of the control group revealed extensive induction and aggregation of Aβ in the cuticle, coelomocytes, and the tail ( **Figure 1E** ). Marked improvements were visible with the naked eye between experimental groups, with moderate reductions of amyloid burden with quercetin 350 µM and rifampicin 75 µM. The combination group exhibited the most visible reduction in fluorescence and Aβ burden. After quantification of the fluorescent images of the induced transgenic *C. elegans* using GreenIntensityCalculator.py (Statzer et al., 2021) and one-way ANOVA analysis, we validated that quercetin 350 µM and rifampicin 75 µM significantly reduced the Aβ burden compared to control + 0.2% DMSO ( *P* values <0.0001 and 0.0002, respectively). Interestingly, the combination therapy of quercetin 350 µM and rifampicin 75 µM reduced Aβ burden compared to control + 0.2% DMSO ( *P* <0.0001), but was more significant than both quercetin 350 µM ( *P* =0.0235) and rifampicin 75 µM ( *P* =0.0023) alone, suggesting that the combination therapy of quercetin 350 µM and rifampicin 75 µM is superior to either drug alone in the clearance of Aβ aggregation in *C. elegans* due to a potential compounding effect of the drugs ( **Figure 1F, Extended Data Figures 1-2, Data Source File 1** ).

In summary, combination therapy of rifampicin 75 µM and quercetin 350 µM proved to be an effective intervention, stronger than either drug alone, to promote the clearance of heat shock-induced sfGFP::Aβ _1-42_ in the extracellular space of *C.* *elegans* . Interestingly, quercetin has been shown to ameliorate Aβ-induced muscle paralysis when under the control of a muscle-specific promoter through upregulation of proteasomal and protein degradation pathways (Regitz et al., 2014) , and rifampicin has been shown previously to reduce advanced glycation end products (AGEs) and increase lifespan through upregulation of *daf-16* in *C. elegans* (Golegaonkar et al., 2015) , potentially implicating another interaction between amyloidosis and the extracellular matrix network, which can be explored further in future experiments.

However, the limitations of this study primarily stem from the use of only one assay, as the target hypothesis was to determine whether these pharmaceutical or nutraceutical compounds would affect sfGFP::Aβ _1-42_ induction, aggregation, and/or clearance. An independent biochemical approach would help bolster claims for the activity of the molecules, including, but not limited to the reduction of aggregation, fibrils, and soluble/insoluble portions. Additional phenotyping of the animals in response to induction and administration of nutraceuticals is further warranted to explore and understand the spectrum of effects of these compounds on amyloid pathology and general healthspan.

Induction through heat shock of sfGFP::Aβ _1-42_ secretion in *C. elegans* has previously been shown to be successful and results in extensive spreading beyond neuronal tissues (Gallrein et al., 2021; Jongsma et al., 2023) . Although, in our previous study (Jongsma et al., 2023) , we did not observe any aberrant physiological traits post-induction upon our one-time, short, and pulse-chase heat shock approach. Other deficits in lifespan may have been due to drug toxicity, as mentioned previously. Further experiments are required to analyze the effects of the drugs on other phenotypic metrics, such as movement assays, lifespan assays, and other related healthspan measures, as is evident in other trials regarding lifespan-extending mutants or other pharmacological interventions (Rollins et al., 2017) .

Furthermore, the use of the heat shock promoter approach to induce aggregation may also contribute to organismal stress, which would subsequently impact lifespan and healthspan-related assays (Ewald et al., 2016) and may itself be an impetus for the secretion of sfGFP::Aβ _1-42_ . Despite this concern, Jongsma et al., 2023 found that heat shock did not affect the lifespan of *C. elegans* with the sfGFP::Aβ _1-42_ *,* embryonic survival, and only minorly affects larval development in response to increased osmolarity (Jongsma et al., 2023) . Cuticle integrity, chemotaxis, and touch habituation were not affected by HS-induced transgene expression either (Jongsma et al., 2023) . Gallrein et al., 2021 succinctly summarize the effects of different transgene localization expressions in various tissues of differing Aβ _1-42_ types and the related effects on healthspan assays and physiological properties. However, the heat shock promoter may serve as an approach that successfully induces sfGFP::Aβ _1-42_ aggregation in extra-neuronal spaces, with quantifications of the aggregate load possible through the presence of GFP signals in affected areas (Gallrein et al., 2021) .

Interestingly, in trial experiments, we observed that worms that did not have traction on the agar and engaged in excessive thrashing and swimming due to the wetness of the plate exhibited much higher visual loads of sfGFP::Aβ _1-42_ . A single lead approach and single metric analysis may be a limiting factor to understanding how the various pharmaceutical and nutraceutical agents affect healthspan and amyloid burden in *C. elegans* . A focus on such agents, however, offers high translatability of compounds to other model organisms and even for human consumption.

Another remaining question regarding whether lead compounds affect transgene expression in these *C. elegans* models warrants further exploration, as quercetin has been shown to affect transgene expression in *E. coli* when response circuits are manipulated **(** Kashiwagi et al., 2021). Although we found no difference in induction levels of sfGFP::Aβ _1-42_ after 24 hours, further assays are required, such as expression levels of the transgene in response to drug administration to analyze the effects of lead compounds on transgene expression. When quantifying fluorescence, no differences were observed between samples of HS-induced worms that received quercetin administration and those that did not, although quercetin has an excitation peak in a similar range to GFP (Nifli et al., 2007) . Further experiments must be conducted with numerous endpoints, such as western blot and PCR, to isolate certain elements of a downstream cascade that may be contributing to amyloid aggregation and increased amyloid burden. Previous experiments have demonstrated the effects of quercetin-mediated longevity in *daf-16* mutant strains (Saul et al., 2008) , and has been shown to ameliorate deficient motility in aged- and heat-stressed *C. elegans* (Sugamara and Sakamoto, 2020). Ayuda-Durán and colleagues explored the effects of quercetin on the insulin/insulin-like growth factor 1 (IGF-1) signaling pathway (IIS) and downstream genes in response to oxidative stress, primarily involving the genes *age-1 , akt-1 , akt-2 , daf-18 , sgk-1 , daf-2* , and *skn-1* (Ayuda-Durán et al., 2019). In these experiments, quercetin was able to salvage *hsp-16.2* responses in aged worms, and greatly bolstered stress response to oxidative stress (Ayuda-Durán et al., 2019). The modulatory effects of quercetin on the IIS may be of interest in these transgenic models expressing sfGFP::Aβ _1-42_ *,* and alterations in the expression of these targets in response to lead compound administration may be a result of or in turn induce changes in HS-induced sfGFP::Aβ _1-42_ expression (Ayuda-Durán et al., 2019).

In conclusion, waste clearance, breakdown, and removal of Aβ in mammals may be important elements of tackling Alzheimer’s disease and developing therapeutics to alleviate the amyloid burden (Uchida, 2022) . Our work suggests a strategy to test a combinatory regime of rifampicin and quercetin with monoclonal antibody therapies to alleviate amyloid β plaques.

## Methods

*C. elegans Strains*

The strain LSD2104 contains an integrated multi-copy transgene that drives the expression via a heat shock promoter to induce and secret sfGFP::Aβ _1-42_ (Jongsma et al., 2023) .

LSD2104 *xchIs015* [pLSD134 P *hsp-16.2* ::ss *Sel1* ::FLAG::superfolderGFP::spacer::humanAmyloidBeta _1-42_ ::let-858-3’UTR; pRF4 *rol-6 ( su1006 )* ].

*Egg Laying Method*

10 cm petri dishes were filled with NGM agar and subsequently seeded with the OP50 strain of *E. coli.* After the plates had completed drying and proper seeding of the bacterial lawn had occurred, 2-3 adult LSD2104 worms were transferred onto the plates. Once eggs were laid, parent worms were sacrificed. The *C. elegans* were maintained at a consistent 15°C, as the strain was highly heat sensitive, and any perturbation in temperature would result in transgene induction since the *hsp-16.2* promoter is a little bit leaky (Ewald et al., 2016) . 7 days after plating produced L4 *C. elegans* , and 8 days after plating produced day-1 adults. The latent maturation of the LSD2104 strain may have been due to the storage of worms at 15°C to maintain transgene integrity.

*Drug Preparation*

Reagents were diluted in either H _2_ O or DMSO to form stock solutions. The stock solutions were then diluted further in H _2_ O to desired concentrations, with a maximum of 0.2% DMSO in the final solution to be administered as the interventional reagent for the amyloid induction trials.

*Heat Shock Protocol*

Once maturation of worms has occurred, 24 hours before heat shock, 250 µL of the desired compound was pipetted onto the surface of the 10 cm plate. Following 24 hours, plates were placed in a 33°C incubator for 3 hours to induce sfGFP::Aβ _1-42_ expression. In contrast to Jongsma et al, 2023, we heat shocked an hour longer to induce a more visible fluorescent aggregation signal compared to 2 hours of induction (Jongsma et al., 2023) ​. Directly after the heat shock was completed, 250 µL of the desired compound was administered again to the 10 cm plate, and culturing plates were placed at 20°C. 24 hours after the initiation of heat shock, another 250 µL of the desired compound was pipetted onto the 10 cm plate as the 3rd and final administration. 24 hours after the final drug application, worms were prepared for fluorescent imaging and quantification. All plates were thoroughly dried to prevent any swimming, which resulted in stressful conditions for the worm, incurring a higher amyloid aggregate load.

*Imaging*

Dissection slides were prepared by pipetting 50 µL of agar onto the slide. 10 µM of tetramisole or levamisole were pipetted onto the agar, and as many surviving *C. elegans* as possible were transferred (Teuscher et al., 2019) . The *C. elegans* were evenly spread on the agar so as to not overlap cuticle boundaries during imaging, which would disrupt the quantification of fluorescent intensity. Fluorescent imaging was performed at 10X magnification using a bright field fluorescence microscope (Teuscher & Ewald, 2018) . Images were captured of each individual worm with the entire *C. elegans* and fluorescent cuticle in the field of view.

*Quantification*

Quantification of fluorescent images was performed utilizing FIJI imaging software and opening the green intensity calculator code GreenIntensityCalculator.py (accessible at github.com/JongsmaE/GreenIntensityCalculator) in ImageJ (Schindelin et al., 2012; Statzer et al., 2021) . Each individual jpeg image was analyzed to produce quantification of fluorescent intensity, number of pixels, and average intensity per number of pixels (I/N). Using the freehand tool, each worm was isolated from the entire image to exclude extraneous signaling that would disrupt the readout by the GreenIntensityCalculator.py program (Jongsma et al., 2023) .

## Reagents

Quercetin: Merck Life Science N.V., Catalog #1592409

Dimethyl sulfoxide (DMSO): VWR International AG, Catalog #23500.260

Purpurin: Merck Life Science N.V., Catalog #229148-5G

Cannabidiol (CBD): Merck Life Science N.V., Catalog #C-045-1ML

Rifampicin: Merck Life Science N.V., Catalog #R3501-5G

Calcium alpha-ketoglutarate (Ca ^2+^ aKG): Merck Life Science N.V., Catalog #75890-25G

Sodium alpha-ketoglutarate (Na ^+^ aKG): Merck Life Science N.V., Catalog #K1875-5G

## Extended Data

Description: Extended Data Figure 1, 2, and Data for Figure 1, Extended Data Figure 1, 2. Resource Type: Dataset. DOI: 10.22002/n4b15-bp311
